# A Complete Response to Pembrolizumab in Malignant Peritoneal Mesothelioma: A Case Report

**DOI:** 10.7759/cureus.52716

**Published:** 2024-01-22

**Authors:** Maria Bairos Menezes, Rita Pedroso de Lima, Inês Dunões, Mariana Inácio, Rui Dinis

**Affiliations:** 1 Medical Oncology, Hospital Espírito Santo de Évora, Évora, PRT; 2 Surgery, Hospital Espírito Santo de Évora, Évora, PRT

**Keywords:** immune checkpoint inhibitors (icis), clinical complete response, interdisciplinary cancer treatment, treatment, pembrolizumab, malignant peritoneal mesothelioma, immunotherapy

## Abstract

Malignant peritoneal mesothelioma (MPeM) is a rare cancer of the peritoneum with a poor prognosis and nonspecific clinical course. We discuss a case of MPeM in a 59-year-old male who presented with abdominal pain and distension, without any known previous asbestos exposure. The diagnosis was made after a second biopsy finally confirmed epithelioid MPeM in an advanced stage with pleural effusion. The patient underwent six cycles of chemotherapy with cisplatin and pemetrexed, experienced disease progression, and was then started on pembrolizumab as a second-line treatment. The patient achieved a complete response after two years of treatment with pembrolizumab and has been disease-free for almost four years with an Eastern Cooperative Oncology Group (ECOG) performance status of 0. Despite the lack of evidence to support the treatment with immunotherapy for MPeM, our case report encourages its use, highlighting its ability to enable a complete response with pembrolizumab with an excellent quality of life.

## Introduction

Mesothelioma is a malignancy of the mesothelium, the serosal membrane that covers and protects the internal organs of the body. Malignant mesotheliomas predominantly affect the pleural mesothelium (50-60%) and can affect the peritoneum in 20-30% of cases [1]. The global incidence of malignant peritoneal mesothelioma (MPeM) is approximately one per four to five million population, while that of all mesotheliomas is one per one million people. Although the association between asbestos exposure and peritoneal mesothelioma is less strong than in the case of pleural mesothelioma, there is a history of asbestos exposure in approximately 50% of the cases [1].

Malignant mesothelioma has three subtypes with different degrees of aggressiveness: epithelioid (75-90% of cases), sarcomatoid, and biphasic. Epithelioid mesothelioma has the best overall prognosis among the malignant subtypes [2]. The clinical presentation of MPeM is often nonspecific, and a definite diagnosis can only be established by histological examination with immunocytochemical procedures, obtained by laparoscopy or open surgery. Pathologically, positive immunostaining for calretinin enhances the accuracy of the diagnosis [2,3].

## Case presentation

A 59-year-old male presented with abdominal pain and distension associated with a 7% weight loss over two months in September 2018. No history of fever, obstipation, diarrhea, nausea, or vomiting was documented. The patient was a truck driver, with a past medical history of hypertension treated with amlodipine (5 mg), type 2 diabetes treated with metformin (1000 mg), hypercholesterolemia treated with simvastatin (20mg), and Pott’s disease at the age of 17 years old managed with the transplantation of a portion of the right tibia into the spine. He had never smoked and had no known previous exposure to asbestos. There was no family history of cancer. Clinical examination revealed moderate ascites, without signs of chronic liver disease. The cardiovascular and respiratory examination was normal. Blood analysis, including full blood count, kidney and liver function tests, inflammatory markers, hepatitis, a viral and autoimmune screens, were all normal (including carcinoembryonic antigen, CA 19.9, and prostate specific antigen).

**Figure 1:**
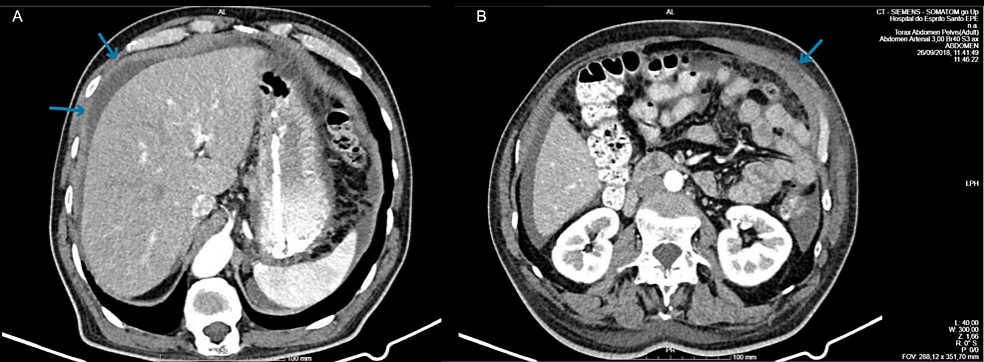
Thoraco-abdominal-pelvic CT performed in 2018 showing moderate ascites with mesenteric densification CT: computed tomography

A heterogeneous echogenic pattern of the liver on abdominal ultrasound was conducted, followed by an abdominal, pelvic, and thoracic CT, which revealed hepatic steatosis, enlargement of the prostate, and moderate ascites with mesenteric densification (Figure 1). Abdominal and pelvic MRI was also performed, which showed the previous alterations. Diagnostic paracentesis was performed and showed chronic inflammatory changes and reactive mesothelial cells, without any signs of infectious peritonitis or malignancy; the serum ascites albumin gradient was low (<1.1 g/dL), and ascitic fluid adenosine deaminase level was also low (6.6 U/L), which excluded tuberculosis.

The patient underwent an upper endoscopy and colonoscopy that did not indicate any alterations. The cardiac ultrasound showed a good systolic global function, subaortic hypertrophy of the ventricular septum, without segmental alterations, and a mild pericardial effusion (posterior pericardial sac). Laparoscopy was performed with a peritoneum biopsy. The samples exhibited mesothelial hyperplasia, without excluding low-grade mesothelioma, and lymphocyte infiltration without granulomas was observed. The screening was negative for mycobacteria (negative Ziehl-Neelsen stain) and fungal infection (negative periodic acid-Schiff stain).

**Figure 2:**
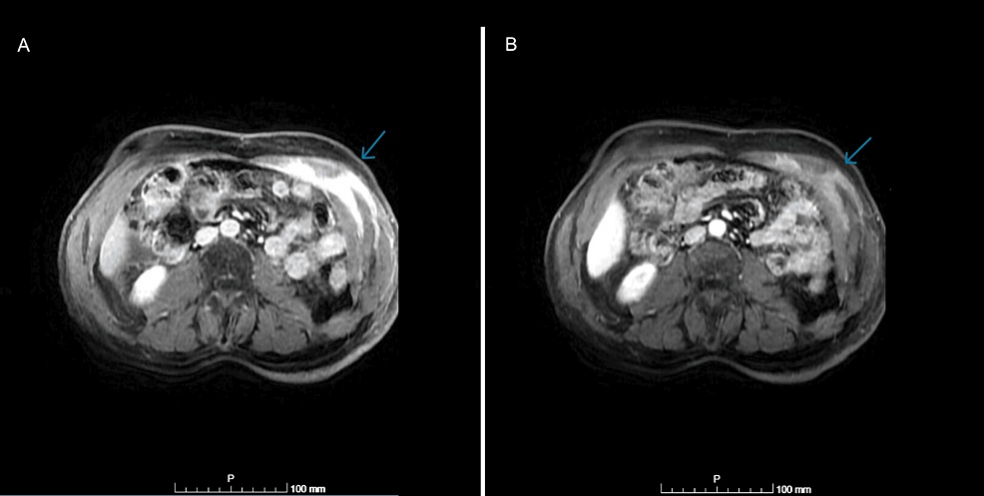
Repeat MRI performed in 2019 showing a thickness in the anterior left abdominal wall MRI: magnetic resonance imaging

The case was discussed with the tumor board of our hospital, as well as with another center due to the rare nature of the condition. Since the patient’s condition eventually improved with pharmacological treatment (non-opioid analgesics and diuretics), with an Eastern Cooperative Oncology Group Performance Status (ECOG PS) of 0-1, it was decided that a repeat MRI scan would be performed in three months. At three months, the abdominal and pelvic MRI demonstrated low/medium ascitic fluid in the subphrenic space and paracolic gut, and an area of thickness in the anterior left abdominal wall, involving the left rectus abdominis and obliques muscles, with an extension of 10.5 cm and a maximum thickness of 2.2 cm (Figure 2). At that time, the patient reported feeling a 1-cm lump in his left flank.

Following the MRI scan, the surgery department performed a biopsy of the palpable abdominal mass in the left flank. This pathological exam confirmed epithelioid MPeM. The immunohistochemical staining was positive for the antibodies vimentin, calretinin, AE1/AE3, CAM 5.2, and cytokeratins 5/6 and 7. A repeat thorax CT scan was performed, which demonstrated right pleural effusion without respiratory insufficiency.

**Figure 3:**
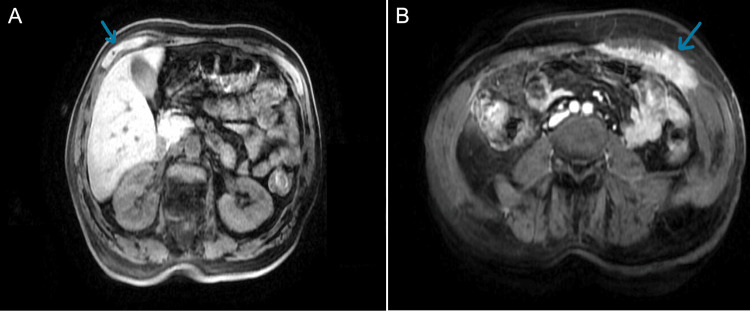
MRI scan after six cycles of chemotherapy showing left lesion in the muscles and a new lesion in the right rectus abdominis muscle (performed in 2019) MRI: magnetic resonance imaging

This case was discussed again with the tumor board, which led to the initiation of chemotherapy due to the patient's advanced stage. Treatment included chemotherapy with cisplatin (75 mg/m^2^) and pemetrexed (500 mg/m^2^) 21/21 days, with folic acid and hydroxocobalamin (vitamin B12). The patient completed six cycles, experiencing nausea and diarrhea grade 2. Afterward, the thoracic CT scan was repeated, which showed maintenance of the right pleural effusion, loculated. Comparatively with the anterior MRI, the patient maintained the left lesion in the muscles with the same dimension and a new lesion in the right rectus abdominis muscle with a maximum thickness of 1.8 cm with an extension of 6 cm (Figure 3), indicative of disease progression.

The case was further discussed with the tumor board, and it was decided to initiate treatment with pembrolizumab. After obtaining approval for this off-label treatment, the patient was started on pembrolizumab (200 mg) every three weeks in January 2020. After four months, the MRI scan was indicative of stable disease. Treatment with immunotherapy was well tolerated, with only hypothyroidism (grade 1) observed. According to a thoracic CT scan, the right pleural effusion was at the same level, while cardiac ultrasound showed the resolution of pericardial effusion.

After one year of immunotherapy, the patient showed a partial partial response on the MRI, and the possibility of radiotherapy was discussed with the tumor board. The patient was not a candidate for surgery. A PET-CT scan was performed, which showed no abdominal lesion with F-fluorodeoxyglucose (FDG) uptake and pleural densification on the right lung with a maximum standardized uptake value (SUV) of 2.03.

**Figure 4:**
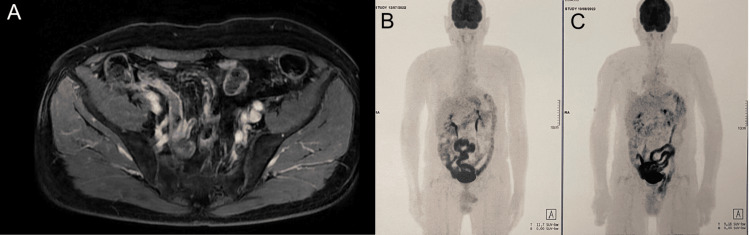
MRI and PET-CT scans performed in 2023 show no evidence of disease MRI: magnetic resonance imaging; PET-CT: positron emission tomography-computed tomography

After further discussion, only immunotherapy was continued. The radiotherapy was deferred given the lack of evidence of disease on the PET-CT scan. In addition, the protocol was changed to pembrolizumab (400 mg) every six weeks. MRI and PET-CT scans performed in 2021 showed muscle fibrosis and no FDG uptake, indicative of a complete response after 18 months of treatment. The patient had to postpone one treatment session because of a mild COVID-19 infection. However, despite this, the treatment was well tolerated with levothyroxine 75 µg (hypothyroidism grade 1). The patient maintained a complete response, as documented on MRI and PET-CT scans performed in 2022 and 2023 (Figure 4). By November 2023, after almost four years of treatment with pembrolizumab, the patient achieved a complete response and has been asymptomatic in the last two years with an ECOG PS of 0.

## Discussion

MPeM is a rare disease that not only presents a diagnostic challenge but has a complex treatment course; it is commonly associated with a poor prognosis. Beyond pemetrexed combination therapy, which is used as a first-line treatment, there is no standard of care for MPeM patients. To address this, immunotherapy has been incorporated into the treatment regimens for patients with several solid tumors. Although pembrolizumab has shown activity in diffuse pleural mesothelioma, scarce data are available concerning MPeM.

In addition, there is currently a lack of prospective trials evaluating the efficacy of immune checkpoint inhibitors due to the small number of MPeM patients treated in clinical trials as well as the exclusion of MPeM from mesothelioma clinical trials. Immune checkpoint inhibitors in peritoneal mesothelioma have been administrated off-label for the treatment of MPeM due to promising outcomes observed in patients with malignant pleural mesothelioma. The optimal benefits of immunotherapy as a first-line therapeutic strategy in pleural mesothelioma have been observed in patients with nonepithelial tumors. However, the majority of MPeM cases are epithelial. Hence, these immunological and molecular differences between pleural and peritoneal mesothelioma hinder the extrapolation of any relevant findings [4,5].

A phase II study evaluated the activity of pembrolizumab in 64 patients who had been treated for mesothelioma with one or two prior regimens of chemotherapy. Of those, only eight patients (12.5%) had MPeM, in which epithelioid was the predominant histology (76.6%). In the study, patients with MPeM had lower overall response rates than patients with pleural mesothelioma. The response rate was reported to be 16% in epithelioid, and it was noted that PDL1 expression was more common in the peritoneal subset with 25% of MPeM classified as having a tumor proportion score (TPS) greater than or equal to 50%. However, whether PDL1 expression is predictive of benefit remains unknown [5-7]. In another study, the clinical efficacy of immunotherapy in patients with advanced MPeM was evaluated, and an overall response rate of 19.2% and a median progression-free survival (PFS) of 5.5 months was reported [8].

Our patient was ruled out as a candidate for surgery by experts in both our department and the surgery department at our center of reference. Radiotherapy was considered, but since the patient achieved a complete response, this modality of treatment was deferred.

## Conclusions

This case report underscores the importance of considering MPeM in the differential diagnosis of ascites, highlighting the complexity of the final diagnosis of this rare condition. Despite the lack of evidence supporting the use of immunotherapy for the treatment of MPeM, we demonstrated that pembrolizumab is a reasonable option as a second-line treatment for MPeM. However, more clinical trials are needed to verify these results. We described a patient who had a complete response after undergoing second-line treatment of MPeM with pembrolizumab, resulting in an excellent tolerance and a high quality of life, despite the aggressive nature of this disease.
